# GP remote consultations with marginalised patients and the importance of place during care: a qualitative study of the role of place in GP consultations

**DOI:** 10.3399/BJGPO.2024.0050

**Published:** 2025-01-15

**Authors:** Ada Humphrey, Steven Cummins, Carl May, Fiona Stevenson

**Affiliations:** 1 The London School of Hygiene and Tropical Medicine (LSHTM), London, UK; 2 Imperial College London, London, UK; 3 University College London (UCL), London, UK

**Keywords:** inequalities, social sciences, qualitative research, remote consultation, primary healthcare, general practitioners

## Abstract

**Background:**

Since the COVID-19 pandemic, there has been an increase in the use of remote consultations in general practice in the UK. This leads to the displacement of the consultation outside of the physical general practice, and its ‘emplacement’ elsewhere, with underexplored consequences for inequities of health care in marginalised groups.

**Aim:**

To examine the place-making demands that remote consultations make on patients, and the ways that these affect their experiences of care, with a focus on the impact on patients from marginalised groups.

**Design & setting:**

Ethnography and interview study (*n* = 15) undertaken at three fieldwork sites in London: a foodbank, a community development organisation, and a drop-in advice centre for migrants. Additionally, GPs (*n* = 5) working at practices in deprived areas of London, Digital Health Hub staff (*n* = 4), and staff at fieldwork sites (*n* = 3) were interviewed.

**Method:**

Ethnographic observation was undertaken for 84 hours at the fieldwork site services, and semi-structured interviews (*n* = 27) took place with service users and service providers. Interviews were conducted in-person and over the phone, and data were analysed through reflexive thematic analysis.

**Results:**

The core themes emerging from the data included challenges securing privacy during remote consultations and the loss of formal healthcare spaces as important places of care. These findings were closely tied to resource access, leading to inequities in experiences of care.

**Conclusion:**

Remote GP consultations are not 'place-less' encounters, and inequities in access to suitable spaces may lead to inequities in experiences of care. Attention should be given to ensuring that patients without appropriate spaces for remote consultations are offered in-person care, or consultation times made more specific to allow for organisation of private space.

## How this fits in

Previous research has demonstrated the potential for remote GP consultations to worsen disparities in healthcare experiences. This study further contributes to this body of research by emphasising how unequal access to appropriate physical spaces for remote GP consultations can pose clinical and safeguarding risks for patients from marginalised groups. It underscores the importance of awareness around patients' resources and determining if they support effective remote GP consultations.

## Introduction

The COVID-19 pandemic brought about a transformation in the delivery of primary healthcare services. While the move towards remote health care in the NHS laid out in *The NHS Long Term Plan* was already underway,^1,2^ the challenge of responding to the COVID-19 pandemic in 2020 provoked a surge in the use of remote consultations, primarily via telephone. Further, access to remote consultations is enshrined in policy, as *The NHS Long Term Plan*,^2^ commits to every patient having the right to be offered digital-first primary care by 2023–2024. This study took place November 2021–April 2022, during this time London had emerged from lockdown and many services were transitioning back to face-to-face engagement. In April 2022, 40% of GP appointments in London were conducted over the telephone.^3^

It is widely acknowledged that there are persistent disparities in healthcare access throughout the UK.^4–6^ However, the implications of increased digital and remote service delivery in primary health care on these inequities are not yet fully understood. While there is optimism that remote health care may alleviate barriers to access, it may also introduce new access disparities and reinforce existing ones.^7–13^ During the COVID-19 lockdowns, the home became the primary space where many patients (and some health professionals) conducted remote consultations. However, as restrictions eased, other more public spaces, became increasingly utilised for consultations. This introduces factors beyond the control of the clinician or patient, such as noise, interruptions, lapses in technology, and a lack of privacy. Understanding the influence of spaces available to patients during remote consultations is crucial for ensuring effective healthcare delivery. The displacement of GP consultations from the consultation room into other spaces raises '*questions as to the implications for place making — or more specifically how actors manage the relations between self, other and place as virtual clinical encounters unfold in their home*'.^14^ All spaces possess distinct physical and social characteristics that either facilitate or hinder certain forms of interaction during remote consultations.^15–17^ This significance of place and its impact on remote healthcare interactions is often overlooked in discussions on remote consultations.

This article reports interview data from patients from marginalised groups, GPs, and community-based charity staff to explore how the loss of the physical consultation room during remote consultations impacts care. By exploring the complex relationship between place, marginalisation, and remote primary health care, this article aims to shed light on the potential barriers faced by marginalised populations in accessing satisfactory remote care.

## Method

In-depth semi-structured interviews were conducted across four participant groups between November 2021 and April 2022.

The four groups were as follows: (i) individuals facing socioeconomic marginalisation (*n* = 15), who were recruited through three East London fieldwork sites, a foodbank, a drop-in centre for migrants, and a community development centre; (ii) staff members at the three fieldwork site services (*n* = 3); (iii) GPs working in deprived areas of London (*n* = 5); and (iv) staff members at Digital Health Hub sites across the UK (*n* = 4).

Digital Health Hubs were set up in 2017 (–2020) by the Good Things Foundation in partnership with NHS Digital and NHS England, as part of the Widening Digital Participation Programme. These hubs, hosted through local charities, were places for people to receive digital skills training and access online services.

While marginalisation manifests in diverse and overlapping ways, this study adopted a fundamental cause approach,^18^ focusing on socioeconomic marginalisation in the sampling strategy. Socioeconomic marginalisation is both a cause and result of other forms of marginalisation, and this guided the recruitment of individuals with diverse characteristics (Table 1). Recruiting from marginalised groups posed challenges related to trust and access to suitable fieldwork sites,^19,20^ necessitating opportunistic strategies. Ultimately, owing to COVID-19 restrictions fieldwork sites were chosen based on factors including the availability of potential responders on site, practical considerations during the pandemic, such as access without using public transport, and negotiation of access with gatekeepers, which is a strategy akin to Kaihlanen *et al*’s approach^21^ when studying challenges faced by vulnerable groups using digital health services in Finland.

**Table 1.** Service user responder demographics (*n* = 15)

Sex	Age bracket in years	Ethnicity	Origin	Resident in the UK	Recruitment site	Other relevant information
M	40–49	White British	Eastern Europe	10–20 years	Foodbank	Recently moved from experiencing street homelessness to hostel
F	30–39	Black British	UK	Always	Foodbank	Recently moved from psychiatric unit to community care
M	40–49	Asian	South Asia	≥20 years	Drop-in centre	English second language
F	30–39	White British	UK	Always	Foodbank	
F	60–69	Black	West Africa	≥20 years	Drop-in centre	Refugee, illiterate
M	40–49	Asian British	Middle East	≥20 years	Drop-in centre	Migrant, low literacy
F	40–49	White British	UK	Always	Foodbank	
F	30–39	Black	East Africa	<1 year	Drop-in centre	Asylum seeker, English second language
F	60–69	Black British	UK	Always	Community hub	Experiencing homelessness cyclically with addiction issues (housed at time of interview)
M	60–69	Black British	UK	Always	Community hub	Experiencing street homelessness at time of interview, with ongoing addiction issues
M	40–49	Arabic	Middle East	<1 year	Drop-in centre	Asylum seeker
F	40–49	White British	UK	Always	Foodbank	
F	60–769	Black British	UK	Always	Community hub	
M	60–69	White British	UK	Always	Foodbank	Resident in supported living
M	40–49	Black British	UK	Always	Community hub	Recently came out of experiencing street homelessness

In addition to interviews, the study includes 84 hours of observational work^22^ at the fieldwork site services where group (i) respondents were recruited.

### Recruitment

The in-person recruitment of individuals facing socioeconomic marginalisation was conducted by the researcher (AH) who spent time at the fieldwork site services, observing interactions, introducing the study to service users, and providing information sheets. Respondents at the foodbank and drop-in centre for migrants were directly recruited by AH, while at the community development hub staff identified interested respondents. Eligible respondents included any users of the fieldwork site services. Recruitment was opportunistic initially, transitioning to purposive sampling for the final interviews to ensure a balance of demographic characteristics. Service staff were recruited at the fieldwork sites, GPs through Twitter (now known as X), and Digital Health Hub staff via publicly available email addresses.

### Data collection

Interviews lasting 1–1.5 hours with group (i) respondents occurred in a private room at the field site. Interviews with groups (ii), (iii), and (iv) were conducted using Zoom, and lasted between 30 minutes and 1 hour. Interview guides were developed based on literature review, informational calls with GPs and social impact charities, and input from academic researchers, ensuring relevance to primary care, digital health, and health inequities. Interviews with group (i) covered accessing primary care, general health, digital device use, and experiences of remote consultations. Interviews with other groups focused on the impact of COVID-19 on service provision, healthcare access for marginalised groups, and the digitalisation of public services. Interviews with group (i) took place first, informing subsequent interviews. All interviews were conducted, audio-recorded, and transcribed verbatim by AH.

### Analysis

Reflexive thematic analysis (TA)^23,24^ was employed to analyse study data. This flexible method allows the construction of themes based on developing patterns of meaning to convey the researcher’s interpretation of the qualitative dataset.^25^ Analytical codes were influenced by theoretical concepts, such as the burden of treatment theory,^26,27^ which helped to conceptualise respondent narratives. Data from service users and providers was analysed together. Reflexive TA involves the following six stages: familiarisation with the data; generating codes; constructing candidate themes; reviewing potential themes; defining and naming themes; and producing the report. AH conducted all analysis using NVivo (version 12).

## Results

### Sample characteristics

Group (i) respondes exhibited a wide range of characteristics associated with marginalisation, often representing multi-marginalisation. Demographics for this group can be found in Table 1, while additional information on the participant sample for staff is shown in Table 2.

**Table 2.** Service provider responders

Participant group	
**Fieldwork service staff (ii)**	**Population served**
	Foodbank: low socioeconomic status
	Drop-in centre: immigrants, refugees, asylum seekers
	Community hub: local community including people experiencing homelessness, and migrants
**GPs (iii**)	**Location of practice**
	Newham
	Tower Hamlets
	Tower Hamlets
	Tower Hamlets
	Lewisham
**Digital Health Hub staff (iv**)	**Population served**
	Older adults with disabilities
	Refugees and asylum seekers
	Older adults
	Refugees and asylum seekers

### Experiences of remote consultations

The core themes emerging from data included unpredictable appointment times and 'phone borrowing' limiting privacy, concerns over the GP’s location and privacy, and formal healthcare spaces as emotional places of care lost during remote consultations.

Patients described being made responsible for creating the physical conditions necessary in which to have an effective consultation. Their accounts emphasised the importance of access to private space. This was tied to experiences of socioeconomic marginalisation: for instance, living in overcrowded households, or having unstable access to private mobile phones. The unpredictability of consultation call times exacerbates this challenge, as it makes it more difficult for patients to organise resources to secure access to private spaces. This can impact on experiences of care in detrimental ways, producing both clinical and safeguarding risks. As well as concerns about their own privacy, patients reported being concerned that their GP may also be consulting from a shared space, meaning they could be overheard.

#### Unpredictable appointment times limit the ability to create privacy

The issue of privacy during remote consultations was a key concern of respondents. The extent to which privacy was a problem was tied to the reason for consulting, as well as the space available and time in which to take a call from the GP.

Respondents reported that owing to wide appointment windows, they may end up taking consultations in places that are not fully private, which can limit willingness to disclose information:

*'I’ve had to do it* [consultation] *in my car driving like whereas normally obviously they ask you quite personal questions about how you’re feeling and I felt like I couldn’t quite answer properly because I didn’t want people to look at me like a lunatic while I’m driving down and sitting in traffic tears streaming down me eyes, and, you know, I mean, I think I felt like I really couldn’t answer properly and honestly.'* (9, Female, 40s, White British, foodbank)

The reason for the consultation can impact how important privacy was, and patients spoke about privacy being particularly important when discussing sexual or mental health concerns. The unpredictability of the timing of remote consultations was spoken about as a key barrier to care, and patients reported feeling paralysed into inactivity, taking half days off work to wait at home for a phone call from their GP. This differs from in-person appointments during which patients go to a physical location for a set time and can make more precise plans.

GPs spoke about the inbuilt unpredictability in remote consultations, arguing that giving exact call times would not be feasible:

*'So again, assumption, when we talk about assumptions, you know, we can’t presume that people can pick up a phone all the time, just like, I feel that it is unfair to say, "I’m definitely going to call you at 10 past eight", because actually, the person that you’re speaking to before, has a problem that takes longer than the 10 minutes, you have to deal with that.'* (20, GP, Newham)

This unpredictability was reported as being a matter of clinical safety, allowing for previous consultations to overrun if necessary. Notably, the reasons why the call windows were longer than the appointment slots for in-person consultations was not clear.

However, GPs did give examples of trying to work around patients’ schedules and agree on specific call times in order to ensure they had access to an appropriate space in which to take a call:

*'A lot of our service users didn’t have private spaces and we would have to be very flexible about when we call people to try and allow that opportunity. I remember we had one particular case of a lady who lived ... she was asylum seeker who was living in a shared house* […] *and had paper-thin walls. You know, she had no privacy in her home at all, and she had lots of very difficult medical issues to talk about. We had to try and coordinate a time when all of her … everyone else in the house was out so that she could take the call and be comfortable to you know, to talk fully.'* (26, GP, Lewisham)

This illustrates how individuals’ physical circumstances can limit their capacity to secure privacy without planning. Other examples were given by GPs of fitting call times around patients’ work breaks as they were unable to take a telephone call without asking permission from a manager.

While there was some awareness from GPs that marginalised patients may struggle to create private spaces for care, there was also surprise at the public spaces in which these patients might take calls from, indicating an assumption that privacy was both an obvious precondition to care, as well as accessible:

*'The number of times I remember doing NHS remote calls and someone’s clearly in an office and probably like an open plan office, so it sounds very noisy, or they’re in a shopping centre or something.'* (26, GP, Tower Hamlets*)*

*'I’m very surprised about, you know, the number of patients who will just take a call on the Tube* [London Underground public transport network]*.'* (23, GP, Tower Hamlets)

#### Phone borrowing and privacy

Another reason why patients may take telephone consultations in non-private spaces is the need to borrow a phone. Many respondents reported unstable access to a phone because of a lost or broken handset, or a lack of funds to buy phone minutes. This can lead to people borrowing or renting devices from others. This was particularly true for respondents who were experiencing street homelessness. Borrowing a phone may lead to the patient taking a call in a shared space with the phone owner or, in more extreme examples, speaking via the phone owner:

*'I have* [had to borrow my mum’s phone to contact the doctor] *I’ve had to shout, they put like the doctors and stuff, she puts them on loudspeaker and I have to shout like I give permission for her to speak on my behalf.'* (5, Female, 30s, White British, foodbank)

On top of privacy during a call, patients were concerned about borrowing a phone to speak to their doctor being a burden as they needed phone access for an extended period owing to the unpredictability of appointment time. It could also lead to them needing to disclose their reason for calling the doctor to the person lending them the phone:

*'Having to say to someone "can I phone the doctor", and immediately when I say to my mum obviously she’s my mum so she’s worried and first thing she says is "what’s wrong" … and you know I have to disclose my medical history to her and I know she’s only worried so obviously I do because she’s my mum you know but urm yeah like I don’t really want to disclose things.'* (5, Female, 30s, White British, foodbank)

GPs expressed concerns about the impact of phone borrowing on privacy and spoke about how they look out for verbal cues that a patient may not be alone, which would be a reason to transition to a face-to-face appointment, demonstrating GP awareness of the issue of privacy and potential need to adapt:

*'Yeah. “Oh, I can’t talk right now”, or if they’ve given you someone else’s mobile, then that might be a red flag, and saying, “Can you call this”, and what we tend to do, what I always do is if the patient’s husband answers. I always say, “where is she?” unless the patient’s there and she’s given consent, or I know them both. I would insist that I speak to the woman on her own if not, I say, “okay, I’ll book you a face-to-face appointment.”'* (25, GP, Tower Hamlets)

As well as unplanned lack of privacy, that is, difficulty securing a private space, GPs expressed concern that a lack of privacy can also be a result of coercion whereby a patient’s partner or family member deliberately wants to limit what they are able to speak about with their GP. This was a particular concern regarding domestic violence.

The problem of being unable to follow-up with patients was also mentioned as a complication of phone borrowing during telephone consultations:

*'We did see that* [phone sharing] *a lot … And we’d often have the difficult situation of trying to follow people up and then realising that the number they’ve given us was not actually theirs.*' (26, GP, Lewisham)

#### Concerns over the GP's location and privacy

As well as struggling to secure privacy, patients also expressed concern that their GP may be somewhere not private, where they can be overheard:

*'The doctor could be doing the same thing. Think "oh it’s just my husband or wife* [listening] *or whatever, it don’t matter" ... so you feel a bit. Well with face-to-face you know that conversation is just between me and them. Do you know what I mean? Obviously like they wrote down it down and they might discuss it with another doctor. But yeah, if they want to go home and discuss it then fair enough, but I’d rather not feel like someone sitting there listening to what I’m saying.'* (9, Female, 40s, White British, foodbank)

This fear was spoken about by several respondents, who felt particularly nervous about speaking to an unknown GP over the phone. GPs also reported being asked by patients where they were consulting from (remotely). While all GPs included in this study spoke about the importance of privacy, and pointed out their own duty to confidentiality, they also discussed holding remote consultations from shared spaces:

*'I do one remote surgery a week. And so, I usually do that, from my breakfast table at home, trying to get my son and my wife to sort of be quiet. You know, I haven’t had any sort of negative feedback about that. And I’ve been, I think, you know, most of the people I know, know that I work here* [GP surgery]*, and I guess they’re sort of, you know, in their heads I’m sort of just working from, you know, my office.*' (23, GP, Tower Hamlets)

The above quote highlights how remote consultations lead to unknowns on both ends of a call, and that both patients and GPs consider where the other is located.

#### Healthcare spaces as emotional places of care

The importance of the GP consultation room as a specific sort of place, beyond simply offering physical privacy was also mentioned as a container for difficult conversations:

*'I think there is something very special about the physical healthcare space, for so long it’s been recognised as this confidential space, which allows people not just to talk about health, but to talk about things that they feel that they can’t talk to anyone else about, because they don’t have anywhere else that’s confidential.'* (26, GP, Lewisham)

The effort to recreate the emotional conditions of the physical consulting space during remote consultations was also mentioned:

*'If you’re a skilled practitioner, you can still create that space in the way that you are, and the way that you ask questions, you can still create that in that space. And that’s what I hope, virtually, with a really skilled practitioner, you can do the same thing over the phone. But that takes a lot of time and experience to do that.*' (26, GP, Lewisham)

The account reflects a perceived need to reproduce the in-person consultation experience during remote consultations, suggesting a perception that the emotional container of a physical consultation room should be replicated regardless of consultation modality.

The findings indicate multiple barriers that may emerge for patients when trying to create effective conditions for remote consultations, which can have knock-on effects on their consultation experience. An effective remote consultation requires certain conditions, which include access to digital technology and connection, and a private space. The ability to secure these conditions within a relatively unpredictable window of time requires predictable access to these resources, which may be more challenging for patients from socioeconomically marginalised groups.

## Discussion

### Summary

The findings of this study indicate that the displacement of the GP consultation outside of the institutional healthcare setting of the GP practice has unintended and possibly under-recognised consequences for patients and GPs. The shifting of responsibility to patients to construct appropriate spaces of care represents a more individualised approach to health care through the removal of the shared common space of the GP practice, which acts as a leveller, and the loss of which may contribute to inequities for certain groups of marginalised patients. Further, unpredictable remote consultation times introduce an added layer of uncertainty into the interaction, making it more difficult for patients to arrange resources to create private space.

### Strengths and limitations

The main strength of this study is that it represents the views of individuals experiencing different forms of marginalisation influencing their health care in different ways. Further, study respondents were recruited from outside of the healthcare service, which meant that people who are potentially low users of the healthcare system, and unlikely to be recruited in that way, were represented in the study. An additional strength is that both service users and providers were interviewed, allowing for a comparison of perspectives. A limitation of the study is that it was primarily conducted in London, so viewpoints of those outside of the capital, for instance in rural areas, who may experience remote consultations in substantially different ways, were not captured. Additionally, while a range of experiences are represented in the study sample, not all groups who are marginalised are represented; for instance, those with physical or learning difficulties who may face unique barriers. Further, respondents were all aged >30 years, which limits generalisability to younger patient groups.

### Comparison with existing literature

This study highlights the challenges that patients, particularly those dealing with socioeconomic marginalisation, face in adapting their environments or schedules for remote consultations. Research on ‘place-making’ during remote healthcare interactions has often focused on chronic condition management, and the use of at-home medical devices.^14,15,28,29^ There has been limited discussion about patients’ access to space during GP remote consultations, especially patients from marginalised groups. GP care may encompass both initial, discrete, and ongoing episodes of care, which have potentially different impacts depending on how remote care is navigated. For instance, in initial and discrete episodes of care, both GP and patient will have less opportunity for habituation of navigating remote consultations through iterative learning processes like those seen in chronic condition management.^30,31^

Unlike the GP consultation room, which provides a relatively standardised experience, remote consultations can expose inequities and variations in patients’ living circumstances. If a lack of private space leads to lower levels of disclosure, this can produce inequitable clinical and safeguarding risks. This builds on research^32,33^ that suggests that remote consultations may increase safeguarding risks for patients by making risks harder for GPs to establish. This study indicates this may be a higher risk among patients from marginalised groups owing to lowered capacity to establish private spaces for care. While GPs described active attempts to create equity and privacy for their patients, demonstrating awareness of the issue, for example, through asking about their privacy levels, or planning call times around patients’ ability to secure privacy, their ability to do so is constrained by patients’ capacity to produce these conditions, which are dictated by their individual circumstances. As outlined in the Results, this can lead to GPs converting to face-to-face appointments, to avoid the risks associated with a lack of privacy.

Similarly, while GPs’ relational skills may go some way towards creating an emotionally contained space during remote consultations, this misses the basic issue of the need for a conducive physical environment on the patient’s side. This indicates a possible disjuncture between the sort of consultation GPs try to create through verbal communication, and the physical realities of the consultation for the patient.

The loss of precise appointment times for remote GP consultations, which lead to patients being called while in workplaces, on public transport, or at home surrounded by others has also been discussed by Rosen and colleagues.^34^ They found that that this can lead to patients withholding or distorting clinical or personal information, as well as potentially leading to safeguarding risks owing to the possibility of abusive partners or family members being present during a telephone consultation. The wide call times given for remote consultations suggests they may be seen as more amenable to unpredictability than in-person consultations. However, accounts by service users suggest that owing to the need to organise time and space to create effective conditions for the remote consultation, predictability is seen as important despite the flexibility of where they take the call from. Further, GPs’ accounts of the necessity for unpredictable call times represents a tension, wherein they are aware of their patient’s need for predictable call times but must balance this with the need to give additional time to patients who require it.

Embedded within discussions of place is the issue of privacy and the blurring of private and public boundaries as health care is moved out of institutional healthcare settings into patients’ personal spaces.^29^ Digital technologies are often presented as bringing public spheres into private spaces, for example, large Facebook communities in a family living room. There has been discussion in telemedicine literature of the home becoming a medicalised space as the quasi-public medical gaze enters the private arena of the home through systems such as telemonitoring.^15,35,36^ However, this study suggests that remote GP consultations may also work in the other direction as patients struggle to produce private conditions for care, emplacing private healthcare discussions into public spaces.

### Implications for practice

The current guidance around remote consultations is primarily around managing risks created by a loss of non-verbal and visual cues during remote consultations. However, there is a lack of recognition about the potentially important role of place during consultations, and how individual circumstances may lead to inequities in experiences of care. While the need to create effective conditions for a remote consultation is a responsibility being passed onto all patients, with the potential to create barriers to care, it may have disproportionate effects on those with less, or less predictable, access to resources. This study’s findings indicate that a person’s capacity to organise resources to ensure private space is related to characteristics associated with marginalisation such as economic deprivation leading to phone borrowing. The study findings indicate that a lack of privacy can have a detrimental effect on the consultation, limiting disclosure of clinically relevant information, which in turn can produce clinical and safeguarding risks. These two findings taken together suggest that people experiencing marginalisation, who struggle to organise resources to create privacy for a remote consultation, may be placed at higher risk.

This study was conducted during the COVID-19 pandemic, during which time the majority of consultations took place remotely, and the consultation landscape has since changed. However, the findings of this study remain clinically relevant not only for remote GP consultations, but also for triage approaches and telephone-booking systems, whereby even if a patient is seen face-to-face, they may still be required to undertake some kind of initial remote encounter.

There are several key policy and practice implications. These include a need for clinicians to be attuned to patients’ whereabouts during a remote consultation, and to ensure that they are offered an alternative in-person consultation if they are unable to secure a private space. Second, the unpredictability of call times was found to be a major barrier to creating privacy, indicating that, as far as possible, call-time windows should be kept narrow, although, as discussed by GPs, this needs to be balanced with clinical risk taking, allowing for calls to run longer if necessary. Building on this, clinicians may want to establish whether patients are able to receive a call in a given window of time and make efforts to book in more exact appointment times for patients who require this certainty.

## Data

Data available upon request via https://datacompass.lshtm.ac.uk/id/eprint/3745/.
